# The Effect of Mother Phubbing on Young Children’s Emotional and Behavioral Problems: A Moderated Mediation Model of Mother–Child Attachment and Parenting Stress

**DOI:** 10.3390/ijerph192416911

**Published:** 2022-12-16

**Authors:** Huan Lv, Wenyu Ye, Suiqing Chen, Hongfeng Zhang, Ruiming Wang

**Affiliations:** 1 Philosophy and Social Science Laboratory of Reading and Development in Children and Adolescents (South China Normal University), Ministry of Education, Guangzhou 510631, China; 2 FoShan University, FoShan 528225, China; 3 School of Education, Guangzhou University, Guangzhou 510006, China; 4 School of Educational Sciences, Lingnan Normal University, Zhanjiang 524048, China; 5 Faculty of Humanities and Social Sciences, Macao Polytechnic University, Macao 999078, China; 6 Center for Studies of Psychological Application, School of Psychology, South China Normal University, Guangzhou 510631, China

**Keywords:** mother phubbing, emotional and behavioral problems, maternal parenting stress, mother–child attachment, young children

## Abstract

Phubbing—the act of ignoring someone physically present in favor of a mobile phone—is increasingly prevalent in families, and mothers’ phubbing behaviors may have a particularly important effect on young children’s development. Accordingly, this study explores the mediating role of mother–child attachment in the relationship between mother phubbing and children’s emotional and behavioral problems, as well as the role of maternal parenting stress in moderating the mediation effect. A total of 988 mothers of young children (mean age = 4.93, SD = 0.94) were surveyed using four scales, and the resulting data was statistically analyzed. The study found that (1) mother phubbing was significantly and positively correlated with children’s emotional and behavioral problems (*r* = 0.19, *p* < 0.01), (2) mother–child attachment mediated the relationship between mother phubbing and children’s emotional and behavioral problems, and (3) the relationship between mother–child attachment and children’s emotional and behavioral problems was moderated by maternal parenting stress. The present study offers fresh evidence of how mother phubbing affects young children’s emotional and behavioral difficulties. The need to reduce maternal parental stress and buffer mothers from its effects are highlighted as vital factors in promoting secure mother–child attachment and alleviating young children’s problems.

## 1. Introduction

Smartphones are now an essential part of many people’s daily lives. The new concept of “phubbing”, which is a portmanteau of “phone” and “snubbing,” refers to the practice of ignoring other people and focusing on smartphones during interpersonal communication [1,2,3]. Whilst prior research has reported phubbing’s negative effects in companies or workplaces [4], personal romantic relationships [5], and the educational field [6], much less is known about its influence on young children’s emotional and behavioral problems (EBPs). Recently, phubbing in the family environment has emerged as a major social issue attracting considerable academic interest. Parental phubbing is a common negative parenting behavior that impacts parent–child relationships and children’s emotions [7,8,9]. However, earlier studies mainly examined the effects of parental phubbing on adolescents without distinguishing between the phubbing behaviors of fathers and mothers [10,11,12]. This overlooks the fact that mothers’ childcare roles differ from those of fathers, and maternal parenting behaviors exert a distinctive influence on the developmental path of young children [13,14]. However, few studies to date have investigated factors, such as mother–child attachment and maternal parenting stress, that may exacerbate or ameliorate the effects of mother phubbing on young children’s EBPs. The present study aimed to address these gaps in knowledge.

### 1.1. The Links between Mother Phubbing and Children’s EBPs

Emotional and behavioral problems in children and adolescents have increased in many countries over the past decade [15,16,17]. When such issues begin in early childhood, they are more persistent than adolescent-onset conduct problems and more likely to recur in later life. Parental acceptance–rejection theory suggests that children require acceptance from their parents [18]. However, when this need is not met and children are subjected to neglect, they are more disposed to behavioral or mental health problems, such as depression [19]. One study found that parental ratings of young children’s behavioral problems were the strongest predictor of antisocial behavior disorders at a 6-year follow-forward assessment [20]. Other research has similarly determined that preschoolers’ externalization of problems predicted greater internalization of issues at ages 10–11 [21].

All in all, young children are in a sensitive period of development that may be hindered by an indifferent environment, uncaring parents, and a lack of interactive language. It is therefore worth exploring the factors influencing young children’s EBPs, since many factors linked to the family play a very important role in the emergence and exacerbation of behavioral problems. There are many negative effects of parental phubbing, such as lowered parenting responsiveness [22] and reduced parent–child interaction quality [23]. In line with previous research, we contend that mother phubbing constitutes a form of rejection, neglect, or indifference to young children that may influence their EBPs. On this basis, we proposed our first hypothesis (H1): Mother phubbing is positively associated with young children’s EBPs.

### 1.2. The Mediating Role of Mother–Child Attachment

Parent–child attachment refers to a lasting and stable emotional connection between an individual and his or her parents through long-term life companionship [24]. To date, a large and growing body of literature has proved that parent–child attachment negatively correlates with young children’s behavioral problems. One systematic review showed that insecure early childhood attachment is positively correlated with behavioral problems in preschoolers [25]. Emotional regulation [26], peer interaction [27], hyperactivity [28], prosocial behavior [29], and various behavioral problems have all been associated—either positively or negatively—with parent–child attachment.

The displacement hypothesis [30,31] claims that time used on media and technology devices may displace real-life interactions and decrease meaningful mother–child connections. For example, a structured laboratory task showed that mothers with a mobile device initiated fewer verbal and nonverbal interactions with their children than mothers with no device [32]. Excessive use of social media has also been shown to affect parenting, causing parental distraction and decreasing levels of everyday engagement from mothers and fathers [33]. Mobile phones interfere with parent–child interactions, making parents less responsive to their children’s needs [34]. Moreover, research on adolescents has found that children who experience more parent phubbing report more alienation [35] and less belonging [2], leading to less secure attachments [36]. Based on these findings, we formulated the next hypothesis (H2): mother–child attachment mediates the relationship between mother phubbing and children’s behavioral problems.

### 1.3. The Moderating Role of Maternal Parenting Stress

Parenting stress is defined as negative feelings towards oneself and one’s offspring, and these feelings are directly attributable to the demands of parenthood [37]. Research emphasizes the importance of assessing parental stress levels and parenting styles when studying and intervening in preschoolers’ behavioral and social competence [38], because higher parenting stress is known to predict lower child health ratings [39,40] and levels of EBPs. Moreover, parenting stress is negatively associated with the parent–child relationship [41].

Although most research on parenting stress and child behavior problems characterizes higher levels of parenting stress as a source of family vulnerability, other research emphasizes that lower parenting stress may be a source of family protection [42]. However, little research has investigated whether low parenting stress buffers the effects of parent phubbing on children’s EBPs. That is to say, depending on its level, parenting stress may be a source of family protection as much as vulnerability to the preexisting risks of EBPs. The cumulative risk model [43] predicts that low parenting stress both secures mother–child attachment and reduces the risk of children’s EBPs. Specifically, it is associated with better cognitive, self-regulatory, and socioemotional development in children [44,45]. Therefore, the present study hypothesizes that the relationships between mother–child attachment and children’s EBPs are moderated by maternal parenting stress (H3).

**Figure 1:**
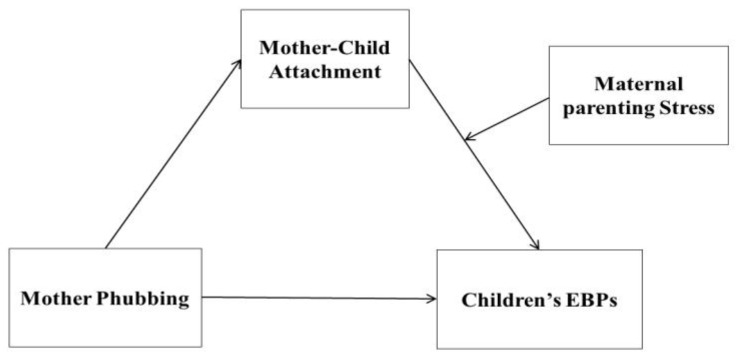
Research hypothesis model.

In this study, we developed a moderated mediation model to explore the mechanisms by which mother phubbing interacts with children’s EBPs (Figure 1). We investigated whether mother–child attachment mediates between mother phubbing and children’s EBPs and whether maternal parenting stress moderates the link between mother–child attachment and children’s EBPs.

## 2. Materials and Methods

### 2.1. Participants

Online questionnaires were distributed on a Chinese online survey platform named Wenjuanxing (http://www.wjx.cn, accessed on 29 March 2021) to survey the mothers of young children in Guangdong province, China. Using convenience sampling, we surveyed 1012 mothers of kindergarten-age children from 29 March to 6 April 2021. Informed consent was obtained from the kindergarten and the children’s parents. The researchers provided free health guidance for parents and children after the questionnaires had been completed. The research was reviewed and approved by the Ethics Committee of South China Normal University (protocol number: 2020-4-068).

In total, 28 invalid questionnaires were excluded because they had been sent from the same internet protocol address, because the children were outside the 3–6 age range, or because the mothers had completed the questionnaires too quickly/slowly, with two standard deviations of completion time as the boundary. This left 988 valid questionnaires—an effective response rate of 97.63%. The children’s ages ranged from 3 to 6 (M = 4.95, SD = 0.88). The young children included 47.98% girls and 52.02% boys. A total of 26.62% of the young children were the family’s only children, and 73.38% were not the only children. Further, concerning mothers’ age, 32.09% of participants were in their twenties, 53.84% were in their thirties, and 14.07% were over their forties. A total of 19.13% of participants were from rural areas, and 80.87% were from urban areas. A total of 57.08% of the mothers were university graduates, followed by 28.44% post-graduates, 9.92% high school graduates, and 4.55% Ph.D. holders.

### 2.2. Measures

#### 2.2.1. The Phubbing Scale

The phubbing scale was used to assess mothers’ phubbing behaviors whilst caring for children. The scale is a revized Chinese version of the generic scale of phubbing [2]. It measures phubbing over 15 items (e.g., “I would rather pay attention to my phone than talk to others”) and consists of four subscales: “nomophobia”, “interpersonal conflict”, “self-isolation”, and “problem acknowledgement”. Each item is assessed on a five-point scale from 1 (never) to 5 (always), with higher scores indicating higher levels of phubbing. The Cronbach’s alpha for the phubbing scale in this study was 0.86.

#### 2.2.2. Parent–Child Attachment Scale

A rating scale of 18 items adapted from the Attachment Q-set [46] was used to assess mother–child attachment. The items primarily measure how the child solicits comfort and support from the mother when emotionally distressed in realistic and hypothetical situations (e.g., “Child is easily comforted by you when distressed”). The mothers’ responses were recorded on a 5-point scale ranging from 1 (totally inconsistent) to 5 (totally consistent), with higher scores indicating higher levels of parent–child attachment. We used the Chinese version [47], and a Cronbach’s α of 0.78 was recorded.

#### 2.2.3. Parenting Stress Index-Short Form

Maternal parenting stress was assessed with the Chinese version of the Parenting Stress Index-Short Form (PSI-SF) [48,49]. The 36-item PSI-SF has three subscales: “parental distress”, “parent–child dysfunctional interaction”, and “difficult child”. The items are rated on a five-point scale, ranging from 1 (strongly disagree) to 5 (strongly agree), with higher scores indicating higher levels of parenting stress. In the present study, the Cronbach’s alpha score for the total scale was 0.93.

#### 2.2.4. Strengths and Difficulties Questionnaire

The EBPs of children aged 3–6 were measured using the mother-reported Strengths and Difficulties Questionnaire (SDQ). The SDQ is a 25-item scale measuring four domains of difficulties (hyperactivity, emotional symptoms, conduct problems, and peer problems) and prosocial behavior. This study focused on the problem subscales: the “prosocial behavior” domain was positive and would have affected the outcome, so it was deleted [50]. The items are rated on a three-point scale ranging from 0 (not true) to 2 (certainly true), with higher scores indicating more severe EBPs. In this study, the Cronbach’s alpha for the Strengths and Difficulties Questionnaire was 0.74.

### 2.3. Statistical Analyses

Statistical analyses were performed using SPSS version 26.0 and the SPSS PROCESS macro [51]. “Gender”, “grade”, and “only child or not” were used as control variables since they have been consistently reported to be crucial predictors of children’s EBPs.

Firstly, we constructed a Pearson correlation matrix that included mother phubbing, mother–child attachment, maternal parenting stress, and children’s EBPs. Secondly, the Bootstrap method (with 5000 resamples) was used to test the mediating and moderating effects. We then tested the mediating effect of mother–child attachment on the link between mother phubbing and children’s EBPs via the SPSS PROCESS (Model 4) macro (Hypotheses 1 and 2) [51]. Thirdly, we investigated the moderating effect of maternal parenting stress on the indirect relationship between mother–child attachment and children’s EBPs with the SPSS PROCESS macro (Model 14; Hypotheses 1, 2, and 3) [51]. All continuous variables were standardized. The bootstrapping technique was used to estimate 5000 resamples of the data to determine whether the effects in Model 4 and Model 14 were significant. Finally, 95% bias-corrected confidence intervals (CIs) were calculated and did not include zero, indicating that the effects were significant.

## 3. Results

### 3.1. Correlations between the Main Study Variables

The results of the Pearson correlation are presented in Table 1 below. The table shows that mother phubbing was negatively correlated with mother–child attachment (*r* = −0.18, *p* < 0.01) and positively with maternal parenting stress (*r* = 0.41, *p* < 0.01) and children’s EBPs (*r* = 0.19, *p* < 0.01). Mother–child attachment was negatively associated with maternal parenting stress (*r* = −0.50, *p* < 0.01) and children’s EBPs (*r* = −0.36, *p* < 0.01) whilst maternal parenting stress was positively related to children’s EBPs (*r* = 0.55, *p* < 0.01). The effect sizes of these correlation coefficients above were small-to-medium, according to Cohen’s standard [52].

### 3.2. Testing of the Mediation Model

The results of the mediation model investigating the relationship between mother phubbing, mother–child attachment, and children’s EBPs are presented in Table 2 below. The variables of “gender”, “grade”, and “only child or not” were used as control variables. Our research adopted the bias-corrected bootstrapping techniques provided by the Hayes PROCESS macro (Model 4) to test the mediation effect [51]. As Table 2 Model 1 (CEPBs) shows, mother phubbing was significantly and positively associated with children’s EBPs (*β* = 0.19, *p* < 0.001, 95% CI [0.13, 0.25]), thus supporting the first hypothesis. According to Model 2 (MCA) and Model 3 (CEPBs), mother phubbing was negatively associated with mother–child attachment to a significant degree (*β* = −0.18, *p* < 0.001, 95% CI [−0.24, −0.12]) and positively linked to children’s EBPs, also at a significant level (*β* = 0.13, *p* < 0.001, 95% CI [0.07, 0.19]). The negative association between mother–child attachment and children’s EBPs remained significant (*β* = −0.34, *p* < 0.001, 95% CI [−0.40, −0.28]). Thus, our second hypothesis, that mother–child attachment significantly mediated mother phubbing and children’s EBPs, was also supported.

### 3.3. Testing of the Moderated Mediation Model

We then tested the moderated mediation model and evaluated the moderating effect of maternal parenting stress on the link between mother–child attachment and children’s EBPs. Controlling for the variables of “gender”, “grade” and “only child or not” using Model 4 (CEPBs; see Table 2) revealed that mother–child attachment was significantly and negatively associated with children’s EBPs (*β* = −0.12, *p* < 0.001, 95% CI [−0.18, −0.06]). The association between maternal parenting stress and children’s EBPs remained significant (*β* = 0.54, *p* < 0.001, 95% CI [0.47, 0.60]), and the product (interaction term) of mother–child attachment and maternal parenting stress exerted a significant predictive effect on children’s EBPs (*β* = 0.07, *p* < 0.01, 95% CI [0.02, 0.11]), suggesting that maternal parenting stress had a moderating role in the relationship between mother–child attachment and children’s EBPs. Specifically, the results indicated that maternal parenting stress could moderate the second half of the indirect pathway, thereby supporting Hypothesis 3.

**Figure 2:**
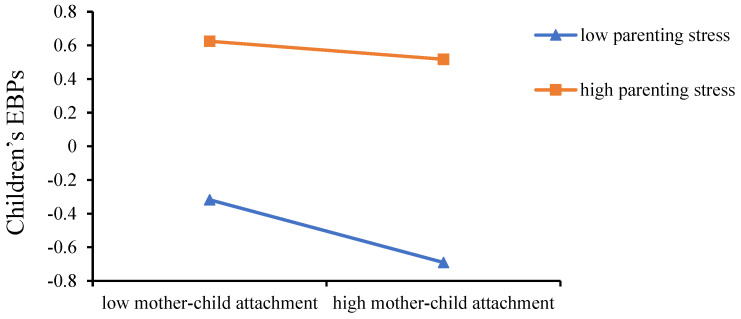
Interaction between mother–child attachment and parenting stress on children’s EBPs.

Our research adopted the Hayes’ PROCESS program to assess the conditional indirect effect [51]. To confirm these interpretations, simple slope tests were carried out to show the effect of mother–child attachment on children’s EBPs at high (+1 SD) and low levels (−1 SD) of maternal parenting stress (see Figure 2). The tests showed that (see Table 3), for mothers with low levels of maternal parenting stress (−1 SD), higher mother–child attachment was significantly associated with lower incidences of children’s EBPs (*β*_simple_ = −0.19, *p* < 0.001, 95% CI [−0.26, −0.11]). However, for mothers whose maternal parenting stress levels were high (+1 SD), the effect of mother–child attachment on children’s EBPs was non-significant (*β*_simple_ = −0.06, *p* > 0.05, 95% CI [−0.12, 0.01]). These results show that maternal parenting stress had a significant moderating effect on the association between mother–child attachment and children’s EBPs.

## 4. Discussion

In this study, we sought to understand how mother phubbing affected young children’s EBPs and the influence of mother–child attachment and maternal parenting stress on this relationship. We found that mothers’ phubbing behavior was negatively associated with children’s EBPs. Moreover, mother phubbing, children’s EBPs, mother–child attachment, and maternal parenting stress formed a moderated mediation model with the potential to inform programs to prevent or ameliorate the deleterious effects of maternal parenting stress on young children’s EBPs.

### 4.1. The Direct Effect of Mother Phubbing on Children’s EBPs

Our research extended prior work by examining the effect of mother phubbing on young children’s EBPs in additional detail. Specifically, we found that mothers’ phubbing behavior was negatively associated with children’s EBPs. The phubbing of kids by mothers or fathers has proved to be a common negative parenting behavior affecting the parent–child relationship and children’s emotions [7,8,9], with its negative impacts resurfacing in older children and adolescents. The psychological state of children aged 3–6 is not as easily perceived by parents because their language expression is insufficiently developed. Mothers using mobile phones may be unable to respond promptly enough to allow the child to establish appropriate behavioral regulation, according to one study [22]. Interview data has further shown that parents struggle to allocate attention between their children and the use of mobile devices for work or social purposes, making it challenging to read and respond to their children’s behavioral and emotional cues [53]. In addition, ecosystem theory shows that the family environment strongly influences the behavioral development of children [54]. Young children’s development is highly sensitive to external conditions and will be hindered by an indifferent environment, uncaring parents, and lack of interactive language, thereby increasing their propensity to behavioral problems.

In the literature review, we contended that mother phubbing is a form of rejection and neglect and that children will take various actions to obtain the phubbing parent’s attention [55]. Our findings support parental acceptance–rejection theory, which suggests that children need acceptance from their parents [18]. Research into patterns of mobile device usage in families demonstrates that children are often frustrated by the sudden withdrawal of parental attention when responding to a notification on a mobile device, especially if the reason for this is unclear [56]. In addition, young children possess weaker emotional regulation skills and often show dissatisfaction through behavioral problems to attract their mothers’ attention. As phubbing is a form of social exclusion [57], rejected children are more inclined to develop behavioral problems. Overall, these results are corroborated by our finding that mother phubbing was significantly and positively correlated with children’s EBPs.

### 4.2. The Mediating Effect of Mother–Child Attachment

The results of the current study showed that mother–child attachment mediated the relation between mother phubbing and children’s EBPs, supporting our initial conjecture that mother–child attachment would be a key predictor of children’s EBPs. Consistent with previous findings [58], we found that mother phubbing harms the attachment between mothers and young children, who would be very likely to experience feelings of neglect. These results also align with earlier studies showing that parents who use their phones during parent–child interactions are less sensitive and responsive [32]. Our findings also indicated that the way mothers treat their children is internalized by preschoolers and used in their daily social interactions. In other words, preschoolers may learn this poor interaction style and come to treat their mothers in the same way, thereby forming insecure parent–child attachments.

We also found a significant negative correlation between mother–child attachment and children’s EBPs. This finding supports Bowlby and Ainsworth’s attachment theory [59], which points to secure attachment as the basis of all relationships and the key to healthy child development. In a relationship characterized by secure attachment, the experiences of parent–child interactions form an inner working model used by the child in later interactions [60]. Our result thus supports calls for mothers to reduce phubbing behavior in order to promote good parent–offspring relationships that promote children’s healthy growth.

### 4.3. The Moderating Effect of Parenting Stress

This study explored whether the mediating effect of mother–child attachment on mother phubbing and young children’s EBPs varied with the level of maternal parenting stress. Maternal parenting stress was found to significantly moderate the relationship between mother–child attachment and young children’s EBPs. The mediating model of the effect of mother phubbing on children’s EBPs through mother–child attachment was further clarified by determining the circumstances in which its influence was stronger. We found that, for mothers who reported lower levels of parenting stress, stronger mother–child attachment was associated with fewer children’s EBPs, but this association was non-significant amongst mothers with higher levels of parenting stress. These results indicate that low levels of parental stress moderate the relationship between mother–child attachment and children’s EBPs and may buffer the effects of mother phubbing on such problems, possibly acting as a source of family protection that safeguards mother–child attachment and reduces problematic behavior. Moreover, the findings corroborate earlier studies showing that parenting stress was positively correlated with the risk of substance misuse amongst adopted children [61]. Research on three-year-old children also found that parent–child attachment was related to parenting stress [62]. Understanding the role of maternal parental stress can help to inform intervention efforts targeted at reducing maternal stress and breaking down the cycle of negative mother–child attachment. Importantly, perceived social support may constitute a protective factor for stressed parents by encouraging them to reevaluate or provide solutions to shield themselves from the effects of stressful circumstances [63]. Taken together, the previous and current findings indicate that marshalling external resources such as social support to reduce maternal parenting stress is conducive to buffering the effects of mother phubbing on children’s EBPs.

### 4.4. Limitations

Whilst this study has elucidated the internal mechanism of mother phubbing and children’s EBPs, it contains several limitations that future studies should seek to address. First, it was based on cross-sectional data, making causal relationships impossible to verify. This shortcoming can be overcome by using longitudinal designs and experimental methods in later research. Secondly, the data collected in this research were obtained through self-reports of the mothers, and social desirability bias could not be controlled. In the future, a combination of questionnaires and experiments should be used to gather data on the subject of phubbing.

## 5. Conclusions

To conclude, the present study offered fresh evidence for exploring how mother phubbing affects EBPs amongst Chinese preschoolers. We found that mother phubbing was significantly and negatively correlated with emotional and behavioral problems in children. The moderated mediation model of this study showed that mother–child attachment mediated the relationship between mother phubbing and children’s EBPs, whilst maternal parenting stress moderated the relationship between mother–child attachment and children’s EBPs. One important practical implication is the need to reduce maternal parental stress in order to secure mother–child attachment and alleviate young children’s EBPs. Mothers should also be able to access support of various kinds to buffer themselves against the negative impacts of parenting stress.

## Figures and Tables

**Table 1:** Descriptive statistics and correlations amongst variables (N = 988).

Variables	M	SD	1	2	3	4
1. MP	2.05	0.55	1.00			
2. MCA	4.17	0.41	−0.18 **	1.00		
3. MPS	1.77	0.50	0.41 **	−0.50 **	1.00	
4. CEBPs	0.40	0.21	0.19 **	−0.36 **	0.55 **	1.00

Note: N = 988. ** *p* < 0.01. Abbreviations: M, mean; SD, standard deviation; MP, mother phubbing; MCA, mother–child attachment; MPS, maternal parenting stress; CEBPs, children’s emotional and behavioral problems.

**Table 2:** Linear regression models.

Predictors	Model 1 (CEPBs)		Model 2 (MCA)		Model 3 (CEPBs)		Model 4 (CEPBs)	
	*β*	*t*	*β*	*t*	*β*	*t*	*β*	*t*
Gender	0.18	2.86 **	−0.03	−0.55	0.17	2.84 **	0.18	3.37 ***
OC	−0.11	−1.51	−0.11	−1.59	−0.14	−2.18	−0.13	−2.16 *
Grade	−0.01	−0.19	−0.03	−0.90	−0.02	−0.53	−0.02	−0.53
MP	0.19	6.05 ***	−0.18	−5.70 ***	0.13	4.30 ***	−0.04	−1.51
MCA					−0.34	−11.41 ***	−0.12	−4.01 ***
MPS							0.54	15.98 ***
MCA × MPS							0.07	3.09 **
*R^2^*	0.05		0.03		0.16		0.33	
*F*	12.00 ***		8.71 ***		36.89 ***		69.87 ***	

Notes: N = 988. * *p* < 0.05, ** *p* < 0.01, *** *p* < 0.001. Gender and OC are set as dummy variables into the regression equation. For gender, 0 is female and 1 is male; For OC, 0 is only child and 1 is not only child. Abbreviations: OC, only child or not; MP, mother phubbing; MCA, mother–child attachment; MPS, maternal parenting stress; CEBPs, children’s emotional and behavioral problems.

**Table 3:** Moderation effect of maternal parenting stress.

Parenting Stress Level	Effect	SE	*t*	*p*	LLCI	ULCI
M − 1 SD	−0.19 ***	0.04	−4.846	0.000	−0.26	−0.11
M	−0.12 ***	0.03	−4.008	0.000	−0.18	−0.06
M + 1 SD	−0.06	0.04	−1.557	0.120	−0.12	0.01

Notes: N = 988. *** *p* < 0.001. Abbreviations: LLCI, lower limit confidence interval; ULCI, upper limit confidence interval.

## Data Availability

The data that support the findings of this study are available from the corresponding author upon reasonable request.
